# Weighing Potential Benefits and Harms of *Mycoplasma genitalium* Testing and Treatment Approaches

**DOI:** 10.3201/eid2808.220094

**Published:** 2022-08

**Authors:** Lisa E. Manhart, William M. Geisler, Catriona S. Bradshaw, Jørgen S. Jensen, David H. Martin

**Affiliations:** University of Washington, Seattle, Washington, USA (L.E. Manhart);; University of Alabama at Birmingham, Birmingham, Alabama, USA (W.M. Geisler);; Monash University and University of Melbourne, Melbourne, Victoria, Australia (C.S. Bradshaw);; Statens Serum Institut, Copenhagen, Denmark (J.S. Jensen);; Tulane University, New Orleans, Louisiana, USA (D.H. Martin)

**Keywords:** *Mycoplasma genitalium*, bacterial drug resistance, natural history, bacteria, sexually transmitted infections, antimicrobial resistance

## Abstract

Since *Mycoplasma genitalium* was identified 40 years ago, much of the epidemiology has been described, diagnostic tests have been developed and approved, and recommended treatment approaches have been identified. However, the natural history remains incompletely understood, and antimicrobial resistance has rapidly increased. This review summarizes evidence published since the US Centers for Disease Control and Prevention 2015 Sexually Transmitted Diseases Treatment Guidelines. Data on sequelae remain insufficient, macrolide resistance is common, and fluoroquinolone resistance is increasing. Potential benefits of testing and treatment include resolving symptoms, interrupting transmission, and preventing sequelae. Potential harms include cost, patient anxiety, and increasing antimicrobial resistance.

## Abstract 2

This systematic review demonstrates increasing antimicrobial resistance and incomplete understanding of the bacterium’s natural history.

*Mycoplasma genitalium* causes 20%–30% of male urethritis cases (*1*,*2*) and has been associated with a 2-fold increased risk for female cervicitis, preterm delivery, and pelvic inflammatory disease (PID) (*3*). It has also been linked to tubal-factor infertility in sero-epidemiologic studies, even after accounting for previous chlamydial infection (*4*,*5*). Despite these strong observational data, relatively few prospective studies of reproductive and perinatal complications of *M. genitalium* exist, and a separate meta-analysis of the association with PID restricted to cohort studies (n = 2) observed a smaller, nonstatistically significant association (*6*). Coupled with this epidemiologic conundrum are substantial treatment challenges. Doxycycline is only 30%–40% effective (*7*), and the efficacy of azithromycin and moxifloxacin has declined substantially (*8*,*9*) since the Centers for Disease Control and Prevention (CDC) 2015 Sexually Transmitted Diseases Treatment Guidelines were written. The reasons for the low efficacy of doxycycline are unclear, but treatment failures after azithromycin and moxifloxacin are caused by the emergence and spread of antimicrobial resistance (*10*). In a recent meta-analysis, detection of macrolide resistance mutations worldwide rose from 10% before 2010 to 51% in 2016–2017 (p<0.0001). Detection of the quinolone resistance–associated mutation most clearly linked to treatment failure (S83I) was 4.3% (*10*), although prevalence as high as 84% has been observed in China (*11*). Among urethritis cases in the United States during 2017–2018, macrolide resistance–associated mutations were detected in 64.4% and quinolone resistance–associated mutations in 11.5% (*1*).

## Methods

This systematic review of the evidence was guided by 2 types of key questions (Table): informational questions outlining data published since the 2015 update to the CDC treatment guidelines and discussion questions outlining how the evidence should inform guidelines. We identified articles published in English since the 2015 treatment guidelines (January 1, 2015–November 30, 2021) by using the search terms mycoplasma AND genitalium OR *M. genitalium* to search PubMed. One investigator (L.E.M.) reviewed abstracts and manuscripts for inclusion. All authors verified included papers. In this article, we focus on new evidence on disease syndromes and antibiotic therapy and the implications for testing and treatment strategies.

**Table:** Key questions for the 2021 CDC Sexually Transmitted Infections Treatment Guidelines Review for Mycoplasma genitalium*

Category	Questions
New evidence	1. What new evidence has emerged on associations between *M. genitalium* and reproductive tract disease syndromes?
	2. What is the prevalence of asymptomatic *M. genitalium* infection in various risk groups (e.g., general population, high-risk cisgender women, high-risk men, MSM)?
	3. What is the current efficacy of currently recommended syndromic therapies for NGU, epididymitis, cervicitis, and PID against *M. genitalium*? Does this differ by sex or by sex of sex partners?
	4. What is the current efficacy of moxifloxacin (400 mg × 7–14 d) against *M. genitalium*? Does this differ by sex or by sex of sex partners?
	5. What is the prevalence of antimicrobial resistance-associated gene mutations among *M*. *genitalium* strains? Does this differ by sex or by sex of sex partners?
	6. What antimicrobials other than azithromycin and moxifloxacin have been studied, either in vitro, or in patients with persisting *M. genitalium* infections and what is their efficacy?
	7. What new *M. genitalium* diagnostic assays are on the horizon and what is the expected timeline for FDA approval of additional assays?
	8. What is the time to *M. genitalium* nucleic acid clearance after therapy?
Discussion	1. Who should be tested for *M. genitalium* (e.g., general population, high-risk cisgender women, high-risk men, MSM)?
	2. Should the recommended empiric therapies for NGU, persistent/recurrent NGU, cervicitis, and/or PID be altered in recognition of the role played by *M. genitalium*? If so, how?
	3. What is the preferred therapy for *M. genitalium* after detection by an FDA approved test? Does this differ by sex or by sex of sex partners?
	4. What is the recommended approach in cases where *M. genitalium* infection persists after treatment with a) doxycycline, b) azithromycin, and c) moxifloxacin?
	5. What is the recommended approach to partner management? Does this differ by sex or by sex of sex partner?
	6. Should a test of cure be recommended after antibiotic therapy for a proven *M. genitalium* infection? Does this differ for symptomatic and asymptomatic persons?
	7. Should antimicrobial resistance in *M. genitalium* be monitored in the United States?

*The summary of the primary evidence that informed the responses to these questions can be accessed in the Tables of Evidence posted on the CDC website: https://www.cdc.gov/std/treatment-guidelines/evidence.htm. CDC, Centers for Disease Control and Prevention; FDA, Food and Drug Administration; MSM, men who have sex with men; NGU, nongonococcal urethritis; PID, pelvic inflammatory disease.

## Results

### Identifying Evidence

We identified 743 papers. Of these, we excluded 150 by title and 195 after abstract review; 398 articles underwent full-text review (Figure). Articles did not distinguish birth sex from gender identity, so we use the terms male or men and female or women.

**Figure:**
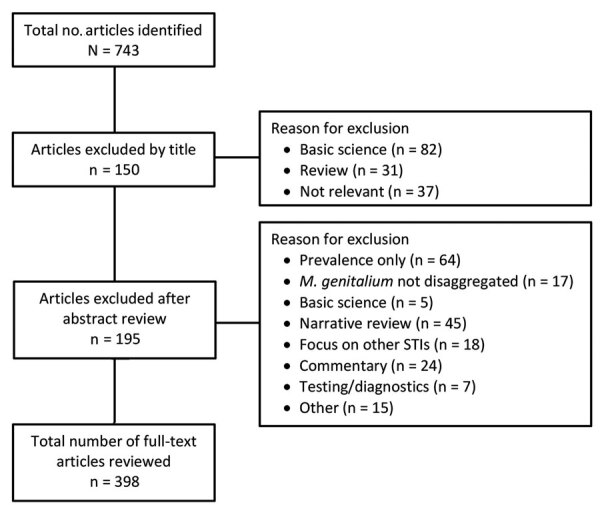
Results of systematic search for literature on *Mycoplasma genitalium* (January 1, 2015–November 30, 2021). STI, sexually transmitted infection.

### Associations with Disease Syndromes

#### Male Urethritis 

*M. genitalium* is consistently and strongly associated with acute, persistent, and recurrent urethritis (*12*) and is an accepted cause of male urethritis. No new data have emerged to change this conclusion.

#### Epididymitis 

Evidence to determine whether *M. genitalium* causes epididymitis remains insufficient. Only case reports have been published (*13*–*15*), and no new studies have compared *M. genitalium* prevalence in men with and without epididymitis.

#### Male Infertility 

Limited available evidence does not support a role for *M. genitalium* as a cause of male infertility. Although an increasing number of studies have investigated this possibility, many evaluated sperm quality rather than documented infertility. Studies enrolling fertile and infertile men have reported no association (*16*–*19*).

#### Proctitis 

Prevalence of rectal *M. genitalium* infection in a recent meta-analysis was higher among men with rectal symptoms than in men without rectal symptoms (16.1% vs. 7.5%; p = 0.039) (*20*). However, few studies included both symptomatic and asymptomatic men (*21*–*24*), and results from those studies are inconsistent. No association was observed between *M. genitalium* and proctitis (odds ratio 0.8; 95% CI 0.45–1.35) in ≈1,000 men who have sex with men (MSM) in Australia (*23*), yet a later study from Australia found MSM with proctitis were significantly more likely to have *M. genitalium* than MSM without proctitis (risk difference 4.3%; 95% CI 1.1%–7.5%) (*24*).

#### Female Cervicitis 

Earlier evidence on the relationship between *M. genitalium* and cervicitis was somewhat conflicting, partly because of varying definitions of cervicitis, but generally supported an association (*3*). This inconsistency remained the case in 4 newer cross-sectional studies. Of those studies, 2 demonstrated increased risk (*25*,*26*), 1 demonstrated increased risk only among a subset of persons (*27*), and 1 reported no increased risk (*28*).

#### Pelvic Inflammatory Disease 

Data remain conflicting on the association of *M. genitalium* with PID, as reflected in the 2 published meta-analyses (*3*,*6*). Studies published since 2014 continued this trend, demonstrating a statistically significant 2-fold increased risk for histologic endometritis (*29*), a nonsignificant ≈3-fold increase in risk for incident PID (*30*), and no association (*31*). No randomized trials testing whether screening and treatment of *M. genitalium* reduces PID incidence have been conducted. In the absence of such trials, there are no population-based screening recommendations.

#### Female Infertility 

In vitro inoculation of *M. genitalium* into fallopian tube tissue results in damage and destruction of cilia (*32*), suggesting that *M. genitalium* might impair female fertility. Consistent with this finding is the 2-fold increased risk for infertility in meta-analysis, especially among studies accounting for other microbial causes of infertility (*3*). More recent studies have reported mixed results. In 2 studies, serologic testing was used to detect antibodies indicative of previous infection; 1 observed a significantly longer time to conception (*33*), whereas the other reported no association with tubal factor infertility (*34*). Three studies used nucleic acid amplification testing (NAAT), which typically detects active infection, and found that *M. genitalium* infection was more common among infertile than fertile women (*35*–*37*).

#### Ectopic Pregnancy 

Data on ectopic pregnancy remain limited. No association was seen among women in Sweden using first-generation serologic assays (*38*), but a study in Saudi Arabia using NAAT to detect *M. genitalium* in fallopian tube tissue demonstrated a 2-fold increased risk for ectopic pregnancy among women (*39*). Additional studies will be required to determine whether *M. genitalium* causes ectopic pregnancy.

#### Preterm Delivery 

Evidence remains insufficient on the relationship between *M. genitalium* infection and preterm delivery. An earlier meta-analysis demonstrated a 2-fold increased risk for preterm delivery, which was stronger among studies accounting for other infections (*3*). However, 2 more recent studies observed low *M. genitalium* prevalence (0%–1%) and were unable to evaluate an association (*40*,*41*). In a third study, *M. genitalium* infection was more common in women in Australia who experienced a preterm birth than in those who did not (15.4% vs. 2.3%), but this difference was not statistically significant (*42*). Adequately powered studies are needed.

#### Other Perinatal Outcomes 

Evidence for an association between *M. genitalium* infection and spontaneous abortion is conflicting. An initial meta-analysis reported significantly increased risk in *M. genitalium*–infected persons (*3*), but subsequent studies did not (*43*–*46*). Using NAAT, *M. genitalium* has been detected in the endotrachea of neonates (*47*), bronchoalveolar lavage samples from children 0–5 years (*48*), and ocular samples from infants born to infected mothers (*49*), suggesting that transmission of *M. genitalium* during vaginal delivery might occur. However, positive NAATs might only reflect residual DNA; larger studies are needed to determine the extent of maternal-to-child transmission.

### Who Should Be Tested for *M. genitalium*?

Screening, diagnostic testing, and tests of cure each have different goals. Screening tests are undertaken in asymptomatic persons to provide treatment, limit sequelae, and prevent transmission. Diagnostic tests are performed in symptomatic persons to direct treatment at a specific pathogen and eliminate the organism. Tests of cure are undertaken to confirm eradication of pathogens. Recommendations for each type of testing are typically grounded in a robust body of evidence. When there is less robust evidence, as with *M. genitalium*, potential benefits must be weighed against potential harms.

#### Screening

Screening at-risk women for gonorrhea and chlamydia is recommended in the United States because infections are frequently asymptomatic (*50*); robust data exist on risk for sequelae from untreated infections (*51*); effective treatment is readily available (*52*,*53*); and, for chlamydia, data from a randomized trial demonstrated decreased risk for PID in persons screened and treated (*54*). *M. genitalium* meets only 1 of these criteria: infections are frequently asymptomatic (≈30%–60% in clinic populations) (*55*,*56*). Data on the risk for sequelae from untreated *M. genitalium* infections in women are less robust than data for chlamydia. Prospective studies of sequelae in women are limited, challenging our ability to infer cause (*3*,*6*). No evidence of adverse sequelae in men exists, although such data are sparse. Given the high rates of macrolide resistance and the advent of fluoroquinolone treatment failures, effective treatment is not readily available for all infected persons (*10*) (see section on antimicrobial therapies). Finally, randomized controlled trials evaluating whether screening and treatment of *M. genitalium* infections could prevent PID and perinatal complications are costly; to date, none have been undertaken despite the articulated need (*57*). Some population subgroups have higher risk for PID, and screening and treatment in those subgroups might yield greater benefit, but this possibility has not been assessed.

##### Benefits of Screening for *M. genitalium*

Screening and effectively treating asymptomatic persons for *M. genitalium* could theoretically reduce transmission and population-level prevalence (*58*). However, reductions in prevalence after implementing screening programs for sexually transmitted infections do not always occur, as demonstrated by increasing chlamydia prevalence in the United States (*59*). This increase has been offset by some declines in PID, although PID rates have slightly increased in recent years (*60*). If identified infections could be effectively treated while minimizing the selection of resistance, screening could reduce the likelihood of transmitted resistance, leading to subsequent reductions in the population-level prevalence of antimicrobial resistance.

##### Harms of Screening for *M. genitalium*

If asymptomatic infections do not cause sequelae, screening and treating will result in unnecessary antibiotic exposure. On an individual level, antibiotics might disrupt a person’s microbiota and lead to other health conditions, and adverse effects associated with antibiotics are occasionally serious (*61*). On a population level, more widespread antibiotic use speeds the emergence and spread of antimicrobial resistance, and multidrug-resistant *M. genitalium* infections are often refractory to treatment. Anecdotal reports suggest that treatment-refractory infections can lead to anxiety and depression that would not occur in the absence of screening. Consistent with earlier assessments (*57*), screening asymptomatic persons for *M. genitalium* is not recommended in the 2021 CDC Sexually Transmitted Infections Treatment Guidelines (*62*).

#### Diagnostic Testing

Etiologic management is increasingly recommended over syndromic management in clinical care (*63*) and requires accurate diagnostic tests. Given the time and difficulty in culturing *M. genitalium* (*64*), NAATs are the preferred method of detection. Two NAATs are approved by the US Food and Drug Administration: the Aptima *Mycoplasma genitalium* assay (Hologic, https://www.hologic.com) and the Cobas TV/MG assay (Roche Diagnostics, https://diagnostics.roche.com). Both tests have good sensitivity and specificity when recommended specimen types are used (urine for men and vaginal swab for women) (*65*). Several other NAATs are available in other countries, including assays that incorporate the detection of macrolide-resistance mutations. The ResistancePlus MG assay (SpeeDx, https://plexpcr.com), S-DiaMGRes assay (Diagenode, https://www.diagenode.com), and RealAccurate TVMGres assay (PathoFinder, https://www.pathofinder.com) are approved for use in Australia, the United Kingdom, Europe, and Canada. To date, no assays to detect macrolide resistance have been approved by the US Food and Drug Administration, although 1 company has developed analyte-specific reagents for use in laboratory-developed tests (Hologic).

##### Benefits of Diagnostic Testing 

The clinical manifestations of *M. genitalium* and *Chlamydia trachomatis* infections are similar (*66*), yet antibiotics recommended for syndromic treatment of urethritis and cervicitis have low efficacy against *M. genitalium* (*67*,*68*). Diagnostic testing for *M. genitalium* when patients first seek care would enable providers to rapidly follow nonspecific syndromic therapy with a regimen more effective against *M. genitalium*. This practice would shorten the time to appropriate therapy, reduce the duration of infection, more rapidly alleviate symptoms, likely reduce risk for sequelae, reduce opportunities for transmission to partners, and lead to fewer interactions with the healthcare system.

##### Harms of Diagnostic Testing 

Diagnostic testing to determine etiology and appropriate therapy might increase healthcare costs. Detecting *M. genitalium* results in additional treatment, potentially with expensive antimicrobial drugs that can have serious adverse effects. Even when symptoms resolve, a positive test result might exacerbate patient distress. Sequelae in men are uncommon, and the debate over sequelae in women has led some to believe asymptomatic *M. genitalium* infections are not of sufficient concern to warrant treatment. If asymptomatic infections truly do not warrant treatment, the cost might outweigh the benefits; however, this possibly has not been studied. The 2021 CDC treatment guidelines recommend diagnostic testing only for persons with persistent or recurrent symptoms (*62*).

#### Tests of Cure

Tests of cure are undertaken to confirm pathogen eradication, preventing transmission and reinfection. Because NAATs detect both viable and nonviable DNA/RNA, explicit time frames for tests of cure have been outlined, ranging from 7–14 days after therapy for pharyngeal gonorrhea to ≈28 days for chlamydia in pregnant persons (*69*). Macrolide-sensitive *M. genitalium* infections are cleared relatively quickly after azithromycin therapy (*70*); when tests of cure are performed, the timeframe is similar to that for gonorrhea and chlamydia. Australian guidelines recommend a test of cure 14–21 days after treatment (*71*).

##### Benefits of Tests of Cure 

The primary benefit of tests of cure is verifying that the organism has been successfully eradicated, which is key for pathogens that cause serious sequelae, particularly during asymptomatic infection. Because *M. genitalium* sometimes recrudesces after symptoms resolve (*72*), a test of cure would identify the need for additional therapy earlier, in turn reducing the risk of infecting sex partners, potentially with resistant strains not detected initially or selected during treatment. Finally, patients might appreciate confirmation that they are cured and not contagious.

##### Harms of Tests of Cure 

A positive test of cure indicates either treatment failure or reinfection. Cases of reinfection are usually retreated with the same antibiotic, whereas cases of treatment failure are typically treated with an alternative antibiotic. When the risk for long-term sequelae is high, the benefit of assuring eradication outweighs the harm of additional antibiotic pressure. When the risk for sequelae is low or uncertain, the potential harm of additional antibiotics might outweigh the benefit of confirming eradication. Despite some evidence that *M. genitalium* can result in adverse sequelae in women, numerous outstanding questions about natural history remain. These questions include uncertainty over the frequency of upper reproductive tract sequelae, frequency of spontaneous clearance, clinical significance of asymptomatic infections, and transmission risk with low organism load that persists after treatment. Given these outstanding questions, the benefit of tests of cure is currently unknown. The 2021 CDC treatment guidelines only recommend tests of cure when resistance testing is not available and moxifloxacin cannot be used (*62*).

### Which Antimicrobial Therapies Should Be Used against *M. genitalium?*

Azithromycin (1 g, 1 dose) was recommended over doxycycline (100 mg 2×/d for 7 days) for *M. genitalium* in the 2015 treatment guidelines. Moxifloxacin was recommended for azithromycin treatment failures (*69*). The 2021 guidelines removed single-dose azithromycin from recommended therapies (*62*), primarily because of increasing antimicrobial resistance (*10*).

#### Doxycycline 

Doxycycline is now the recommended first-line therapy for nongonococcal urethritis and cervicitis in the United States (*62*) and in many other countries globally. However, microbiologic cure rates of *M. genitalium* infection after doxycycline treatment are low, ranging from 30% to 45% in 3 randomized trials of urethritis treatment (*67*,*68*,*72*). Little data are available for women, and no new trials have been performed. Estimates of doxycycline efficacy remain unchanged.

#### Azithromycin 

The efficacy of azithromycin for *M. genitalium* infection declined over time through 2015 (*8*), and more recent studies observed microbiologic cure rates as low as 52% (*73*). Selection for macrolide resistance typically occurs in ≈10%–12% of *M. genitalium*–infected persons receiving single-dose azithromycin therapy (*73*–*76*), and this regimen is no longer recommended in most contexts (*71*,*77*). Some studies have evaluated extended-dose azithromycin (1.5 g given over 5 days) and a meta-analysis suggested less frequent selection of macrolide resistance with this regimen (*78*). However, a historical comparison reported no difference in efficacy or in selection for macrolide resistance between the 2 regimens (*73*). Presence of macrolide-resistance mutations in the 23S rRNA gene is strongly correlated with microbiologic treatment failure after azithromycin, and the global prevalence of these mutations increased from 10% before 2010 to 51% in 2016–2017 (*10*). In 6 US sexual health clinics, the prevalence of macrolide resistance was 64% (range 59.6%–75.9%) and higher in MSM (75.7%) (*1*).

#### Moxifloxacin 

Although the efficacy of moxifloxacin for *M. genitalium* was initially high (100%), it declined to 89% in studies during 2010–2017 (*9*). Sitafloxacin, a more potent fluoroquinolone, has somewhat higher cure rates but is not available in many countries including the United States. Multiple mutations in the *parC* gene have been reported, but only some have been correlated with moxifloxacin treatment failure. The S83I mutation is most common and most strongly associated with treatment failure; it has been detected in 4.3% of infections globally (*10*). Additional mutations in the *gyrA gene* (M95I and D99N) likely have a synergistic effect (*79*), but GyrA mutations are rarely assessed.

#### Resistance-Guided Therapy 

Where diagnostic assays that can detect macrolide resistance mutations are available, sequential resistance-guided therapy is feasible and results in higher cure rates. Under sequential resistance-guided therapy, initial empiric treatment with doxycycline is followed by a second antibiotic (either azithromycin or moxifloxacin) on the basis of the pathogen’s susceptibility profile. Tetracycline resistance in *M. genitalium* develops infrequently, and treatment with doxycycline reduces the organism load, subsequently limiting development of resistance to the second antibiotic (*80*). In Australia, where this approach was developed, macrolide-susceptible infections are treated with high-dose azithromycin (1 g, then 500 mg/d for 3 days) after the initial doxycycline regimen, whereas macrolide-resistant infections are treated with moxifloxacin (400 mg/d for 7 days) after the initial doxycycline regimen. In a setting where macrolide resistance was high (>70%) and quinolone resistance moderate (13%–22%), cure rates for doxycycline followed by azithromycin were 95.4% and 92.0% for doxycycline followed by moxifloxacin; selection for macrolide resistance occurred in only 4.6% (*81*). Given high macrolide resistance in the United States, the 2021 CDC treatment guidelines recommend resistance-guided therapy where possible and sequential treatment with doxycycline followed by moxifloxacin when resistance testing is not available (*62*).

#### PID Treatment 

Recommended outpatient PID treatment in the 2015 CDC treatment guidelines consisted of antimicrobial drugs for empiric treatment of gonorrhea and chlamydia (a cephalosporin and doxycycline). This regimen had limited effectiveness against *M. genitalium*; cure rates were as low as 56% (*69*,*82*). More recent data demonstrated cure rates of ≈95% for PID treatment regimens that incorporated metronidazole, regardless of whether the regimen included azithromycin or moxifloxacin (*83*). Similarly, persistent cervical *M. genitalium* infections were significantly less common among patients receiving a PID regimen with metronidazole than in patients not taking metronidazole (*84*). Metronidazole targets anaerobes and is thought to lack activity against *M. genitalium* (*85*), suggesting that eradicating anaerobes might enhance *M. genitalium* clearance. However, this hypothesis has not yet been evaluated.

#### Alternative Antibiotics 

Minocycline (100 mg orally 2×/d for 14 days) and pristinamycin (1g 3×/d for 10 days) in combination with doxycycline (100 mg 2×/d for 10 days) have been used to treat *M. genitalium*–infected patients when other antibiotics have failed. Minocycline cured 71% (25/35) patients in a sexual health clinic in Australia, and pristinamycin cured ≈75% (55/73) patients (*86*). Minocycline is widely available, but pristinamycin is not in many areas, including the United States. MICs for lefamulin are low (*87*), but no efficacy data for *M. genitalium* infection in humans have been published. Gepotidacin also has low MICs and in vitro experiments suggest combination therapy with doxycycline might improve efficacy, but this possibility has not been tested in vivo (*88*). Longer durations of doxycycline have not been tested in comparative studies, but a recent study found 59% of patients with macrolide resistant infections experienced microbiologic cure after a 14-day regimen (*89*). Combination therapy using doxycycline and moxifloxacin yielded microbiologic cure rates equivalent to those observed using sequential therapy (*90*), suggesting efficacy but no advantage over resistance-guided therapy. Combination therapy using doxycycline and sitafloxacin has been more effective (*91*).

### Sex Partner Management

Concordance of *M. genitalium* infection was 40%–50% in heterosexual partnerships and 27% in MSM partnerships in a recent meta-analysis (*6*). The lower observed concordance in MSM may reflect inclusion of persons tested at only one anatomic site. When both partners were tested at the urethra and rectum, concordance was 42% (*92*). This result suggests that partners of *M. genitalium*–positive patients should be tested and treated to reduce the risk for reinfection. This strategy is recommended in guidelines from Europe, the United Kingdom, and Canada (*93*–*95*), although testing of partners is not specified in Canada. Australia guidelines also recommend contact tracing for heterosexuals and ongoing partners of symptomatic MSM (*71*). No evidence suggests that partner infections have differential antimicrobial susceptibility. The 2021 CDC treatment guidelines recommend testing and treating infected partners with the same antibiotic provided to the index patient (*62*).

## Conclusion

*M. genitalium* is now an established sexually transmitted infection that poses substantial challenges for developing optimal testing and treatment approaches. Antimicrobial resistance has grown rapidly, and untreatable infections have begun to appear. New antimicrobial drugs and better antimicrobial stewardship of existing antibiotics are urgently needed. Resistance-guided therapy is a vital tool to reduce antibiotic pressure and maintain the efficacy of existing antimicrobials. However, only some diagnostic tests incorporate the detection of antimicrobial resistance, limiting our ability to use this tool. Concerns about the rapid spread of resistance have led to recommendations to limit testing and treatment for *M. genitalium* infections to patients with symptoms. Rigorous and adequately powered clinical trials of screening and treatment of *M. genitalium* in women are critically needed. Without a better understanding of the natural history of infection, particularly the risk for sequelae, the benefits and harms of testing and treatment approaches cannot be truly weighed. 
